# Characterization and Machine Learning-Driven Property Prediction of a Novel Hybrid Hydrogel Bioink Considering Extrusion-Based 3D Bioprinting

**DOI:** 10.3390/gels11010045

**Published:** 2025-01-07

**Authors:** Rokeya Sarah, Kory Schimmelpfennig, Riley Rohauer, Christopher L. Lewis, Shah M. Limon, Ahasan Habib

**Affiliations:** 1 Sustainable Product Design and Architecture, Keene State College, Keene, NH 03431, USA; rokeya.sarah@keene.edu; 2 Manufacturing and Mechanical Engineering Technology, Rochester Institute of Technology, Rochester, NY 14623, USA; kms6060@g.rit.edu (K.S.); cllmet@rit.edu (C.L.L.); 3 Biomedical Engineering, Rochester Institute of Technology, Rochester, NY 14623, USA; rbr9577@g.rit.edu; 4 Industrial & Systems Engineering, Slippery Rock University of Pennsylvania, Slippery Rock, PA 16057, USA; shah.limon@sru.edu

**Keywords:** bioink viscosity, predictive modeling, extrusion-based bioprinting, machine learning, rheology, hydrogel composites

## Abstract

The field of tissue engineering has made significant advancements with extrusion-based bioprinting, which uses shear forces to create intricate tissue structures. However, the success of this method heavily relies on the rheological properties of bioinks. Most bioinks use shear-thinning. While a few component-based efforts have been reported to predict the viscosity of bioinks, the impact of shear rate has been vastly ignored. To address this gap, our research presents predictive models using machine learning (ML) algorithms, including polynomial fit (PF), decision tree (DT), and random forest (RF), to estimate bioink viscosity based on component weights and shear rate. We utilized novel bioinks composed of varying percentages of alginate (2–5.25%), gelatin (2–5.25%), and TEMPO-Nano fibrillated cellulose (0.5–1%) at shear rates from 0.1 to 100 s^−1^. Our study analyzed 169 rheological measurements using 80% training and 20% validation data. The results, based on the coefficient of determination (R2) and mean absolute error (MAE), showed that the RF algorithm-based model performed best: [(R2, MAE) RF = (0.99, 0.09), (R2, MAE) PF = (0.95, 0.28), (R2, MAE) DT = (0.98, 0.13)]. These predictive models serve as valuable tools for bioink formulation optimization, allowing researchers to determine effective viscosities without extensive experimental trials to accelerate tissue engineering.

## 1. Introduction

Bioprinting is an evolving technology that utilizes computer-controlled 3D printing to create scaffolds for tissue engineering. According to the American Society for Testing and Materials (ASTM) standards [1], the most common bioprinting techniques include extrusion-based methods (e.g., microextrusion, direct writing) [2,3], jetting-based methods (e.g., inkjet, laser-assisted) [4,5,6], and vat polymerization techniques like stereolithography (SLA) [7,8]. These methods involve the spatial deposition and polymerization of cell-laden bioinks, enabling scaffold-based bio-manufacturing. Bioprinting typically consists of three stages: (i) pre-processing, involving material preparation; (ii) processing, through precise bioink deposition; and (iii) post-processing, which provides temporary support and fosters cell-to-tissue growth [9]. Among those techniques [10,11,12], the extrusion-based one is particularly versatile and capable of depositing a wide range of substances, including various types of bioink [13,14]. By adjusting printing parameters, extrusion-based bioprinting can produce scaffold structures using both acellular biomaterials [15,16,17] and bioink (living cells mixed with hydrogel) [2,18,19], ensuring user-defined geometry. Research indicates that differences in the size and shape of pores in 3D-printed scaffold structures impact cell behaviors [20]. However, when employing extrusion-based bioprinting, there is often a significant gap between the intended design and the actual printed scaffold due to the lack of proper material selection aligning effective printing process properties [21,22]. This poses challenges in achieving precise shape fidelity, biocompatibility, and mechanical integrity in the scaffold. Scientists are currently exploring the optimal biomaterials for creating controlled 3D porous structures through additive manufacturing.

To achieve the defined printability, shape fidelity, and biocompatibility of any proposed bioinks (biomaterials mixed with living cells), various characterization tests have been conducted such as rheological [23,24,25,26], diffusion and collapse [27,28], microstructural [29], and cellular activities (viability, proliferation, and differentiation) [30]. Achieving high-resolution precision and shape fidelity is crucial when fabricating constructs that accurately replicate the structure and architecture of specific tissues or organs [31]. The bioink used in this process must perform a series of complex functions, including safeguarding cells during and after printing, enabling precise control during the printing process, maintaining the structural integrity and physiological environment of the printed cellular constructs, and fostering cell growth and functional tissue formation at high densities. These requirements necessitate a bioink that is both spatially and temporally responsive to stimuli. To meet these multifaceted demands, it is essential to combine various biomaterials, commonly known as hybrid hydrogels [32,33], creating a multifunctional bioink that offers desired biocompatibility, a bioactive microenvironment, shape fidelity, and printability. Such a comprehensive approach is vital for successful tissue engineering applications.

Therefore, various percentages of each element were used, maintaining the overall solid content (8%) to explore the effect of each element based on printability, microstructure, and biocompatibility performances. Alginate, a natural polysaccharide derived from brown algae, provides excellent printability [14], while gelatin, a denatured form of collagen, offers cell-friendly environments [15]. The surface of Nano-fibrillated cellulose (NFC), a derivative of cellulose gel, is altered by oxidation using 2,2,6,6-tetramethylpiperidine-1-oxyl (TEMPO) [34] to add negatively charged carboxylate ions, which is known as TO-NFC, to improve uniformity, dispersibility, homogeneity, and printability (TEMPO-NFC). In our earlier work, it was proposed that novel bioink preparation with alginate, carboxymethyl cellulose (CMC), and TONFC demonstrates the capability of 3D printing scaffolds, ensuring defined geometry and promising cell survivability [35,36]. This motivates us to select these two biomaterials. Moreover, being thermos-sensitive and biologically supportive, gelatin has been extensively used for bioprinting purposes to improve geometrical and cellular activities [37,38,39]. This further reinforced our decision to select gelatin as the third component of the bioink to harness the combined benefits of all three materials. To the best of our knowledge, we are the first to introduce a novel bioink formulation comprising alginate, gelatin, and TO-NFC in this paper. While previous efforts have explored incorporating various fibers into alginate–gelatin compositions to enhance scaffold mechanical properties [17,18], our approach leverages the collective advantages of all three components, aiming to achieve precise scaffold geometry and improved cell viability during the printing process. Finally, Human Mesenchymal Stem Cells (hMSCs) were mixed to prepare bioink, and the cell survivability was observed after seven incubation days [40,41]. In the future, this research group aims to achieve differentiation into bone tissue [42].

We hypothesized that various percentages of hydrogel mixes and shear rates could result from required rheological properties suitable for the extrusion-based bioprinting process. Current bioink optimization practices rely on extensive experimentation with hybrid hydrogel constituents to assess printability, shape fidelity, and biocompatibility. This approach is time-consuming, resource-intensive, and may yield suboptimal results due to the complex solution space. Predicting bioink properties is challenging due to polymer characteristics and chain entanglements [43]. While simple models like Einstein’s linear prediction [44] can work for dilute and Newtonian fluids, and the Cross model can predict viscosity at different shear rates for non-Newtonian fluids [45], these models have limitations. They assume homogeneity and continuity, which may not apply to heterogeneous hybrid hydrogel systems. Additionally, determining rheological factors (n and K) for complex systems can be difficult and require extensive testing. In biomanufacturing, specifically for 3D bioprinting, predicting viscosity across different compositions is more valuable than predicting it for a single composition at various shear rates [46,47]. Existing models are inadequate for this purpose, especially for heterogeneous bioink compositions. Therefore, developing a viscosity prediction tool for bioinks prepared with hybrid hydrogels is crucial to accelerate the complex process of bioink development in the bio-manufacturing research community.

Machine learning (ML) algorithms offer exciting opportunities to enhance all three stages (pre, during, and post) of the bioprinting process, potentially revolutionizing tissue engineering applications, regenerative medicine, and digital bioprinting [48,49,50,51]. Predictive modeling techniques encompass a range of methods, including linear regression analysis [52], support vector regression algorithms [53], and k-nearest neighbor regression [54] approaches. These powerful tools have been applied in various aspects of bioprinting research. ML was used to identify key rheological properties affecting the printing quality of Type I collagen. Using inductive logic programming (ILP), researchers found that high storage modulus and low yield stress were dominant factors [49]. Multiple regression analysis created a simplified linear model to predict printability [49]. Another predictive model for polymer nanocomposite (PNC) viscosity was developed by combining machine learning with nonequilibrium molecular dynamics (NEMD) simulations. This computational framework calculated viscosity under various conditions (shear rates, nanoparticle loadings, and temperatures) using NEMD [55].

Very few of the reported articles considered the constituents’ weights and shear rate simultaneously as a function of predicting viscosity. Moreover, a comparison of viscosity prediction performances of various ML algorithms can help choose the effective one. In this paper, a machine learning framework has been utilized to predict the viscosity of bioink prepared with hybrid hydrogels, aiming to enhance extrusion-based bioprinting techniques. The proposed approach incorporates a series of machine learning algorithms such as polynomial fit, decision tree, and random forest algorithms to determine the viscosity relating to the bioink constituents’ weights and the shear rates. Polynomial fit, decision tree, and random forest were selected because they provide robust and effective predictive modeling capabilities for bioink viscosity [56,57,58]. Table 1 summarizes the features, advantages, and challenges of three ML algorithms used in this article along with a description using the results we observed. These algorithms allow for the simultaneous consideration of key factors, such as bioink formulation and shear rate, which are critical for enhancing extrusion-based bioprinting techniques. By comparing the predictive performance of multiple algorithms, we aim to identify the most accurate and efficient method for viscosity prediction, ultimately improving printability and advancing bioprinting applications.

## 2. Results and Discussion

### 2.1. Rheological Properties of the Hybrid Hydrogels

#### 2.1.1. Flow Behavior of Various Hydrogels

**Figure 1:**
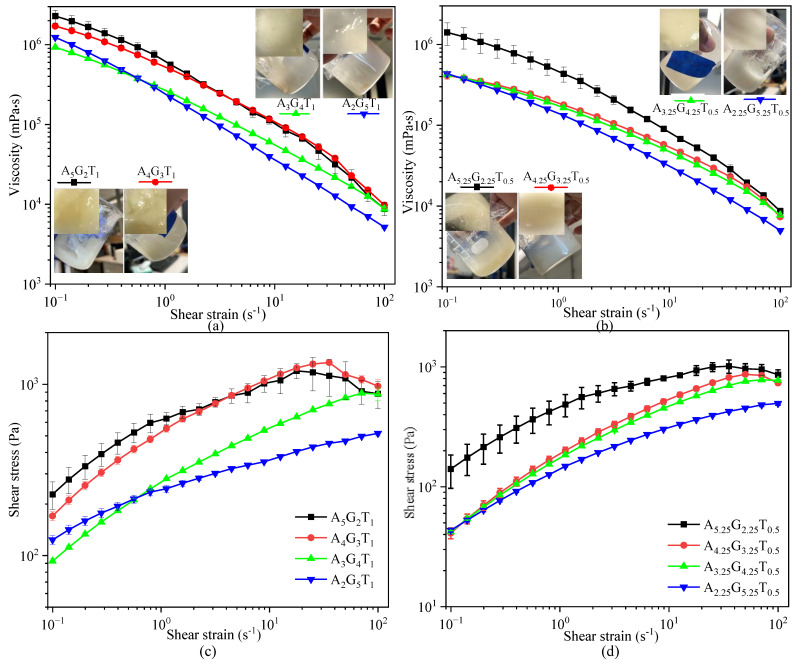
Viscosity of various percentages of alginate and gelatin infilled with: (**a**) 1% TO-NFC and (**b**) 0.5% TO-NFC; shear stress of various percentages of alginate and gelatin infilled with: (**c**) 1% TO-NFC and (**d**) 0.5% TO-NFC.

Under a constant total solid content of 8% and fixed TO-NFC levels of 0.5% and 1%, all formulations with varying alginate and gelatin concentrations demonstrated shear-thinning behavior, as viscosity decreased with increasing shear rate (Figure 1a). While A_5_G_2_T_1_ showed the highest viscosities, A_2_G_5_T_1_ showed the least. A_2.25_G_5.25_T_0.5_ exhibited the lowest viscosity, while A_5.25_G_2.25_T_0.5_ demonstrated the highest. At a fixed TO-NFC concentration (0.5% or 1.0%), alginate content primarily influenced the shear rate. Figure 1a,b clearly illustrates that increasing gelatin concentration reduces viscosity across all compositions as the shear rate increases. Notably, A_2_G_5_T_1_ experienced a significant drop in viscosity, surpassing A_4.25_G_3.25_T_0.5_ at 2.24 s^−1^ shear rate and closely approaching A_2.25_G_5.25_T_0.5_ at 100 s^−1^ shear rate. Furthermore, comparing T_1_ and T_0.5_ compositions revealed that T_1_ formulations consistently exhibited higher viscosities and shear stress than their T_0.5_ counterparts.

Figure 1c,d represents the change in shear stress relating to the shear rate, where the increasing trend proves the shear thinning behavior of those compositions. In the following section, the rheological factors such as $\eta_{\infty }$, $\eta_{0}$, *t*, *m*, *n*, and *K* were determined by fitting the viscosity versus shear rate data to the Cross model and shear stress versus shear rate to the Herschel–Bulkley model.

#### 2.1.2. Determination of Rheological Factors Fitting to Cross and Herschel Models

**Figure 2:**
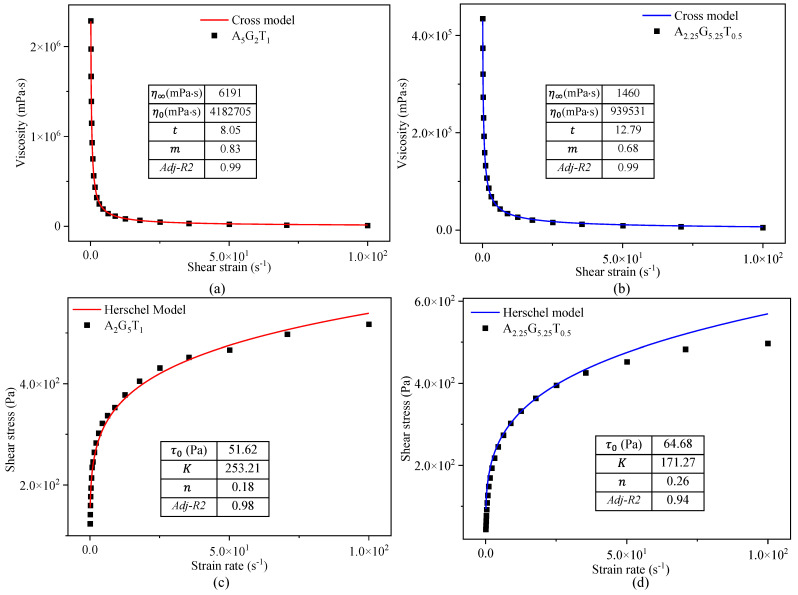
Fitting Cross model to determine $n_{\infty }$, $n_{0}$, t, and m for (**a**) A_5_G_2_T_1_ and (**b**) A_2.25_G_5.25_T_0.5_; fitting Herschel model to determine $\tau_{0}$, K, and n for (**c**) A_2_G_5_T_1_ and (**d**) A_2.25_G_5.25_T_0.5_.

The rheological factors including $\eta_{\infty }$, $\eta_{0}$, *t*, *m*, *n*, and *K* were obtained by fitting the viscosity–shear rate data as shown in Table 2. To demonstrate the fitting of experimental data to the Cross and Herschel–Bulkley model, a total of four compositions such as A_5_G_2_T_1_, A_2_G_5_T_1_, A_4.25_G_3.25_T_0.5,_ and A_2.25_G_5.25_T_0.5_ were considered as shown in Figure 2. The graphs demonstrate that the experimental data for viscosity and shear stress exhibited a strong statistical fit to the Cross and Herschel–Bulkley model, with an adjusted R^2^ value exceeding 0.90 for all compositions. The viscosity versus shear rate data showed an even better fit for both compositions prepared with 0.5% and 1.0% TO-NFC with an adjusted R^2^ value close to 0.99. Table 1 clearly shows that the zero-shear viscosity, zero-shear stress, *K*, *t*, and *m* mostly changed with respect to the concentration of alginate.

#### 2.1.3. Three-Point Interval Thixotropic Test (3iTT)

**Figure 3:**
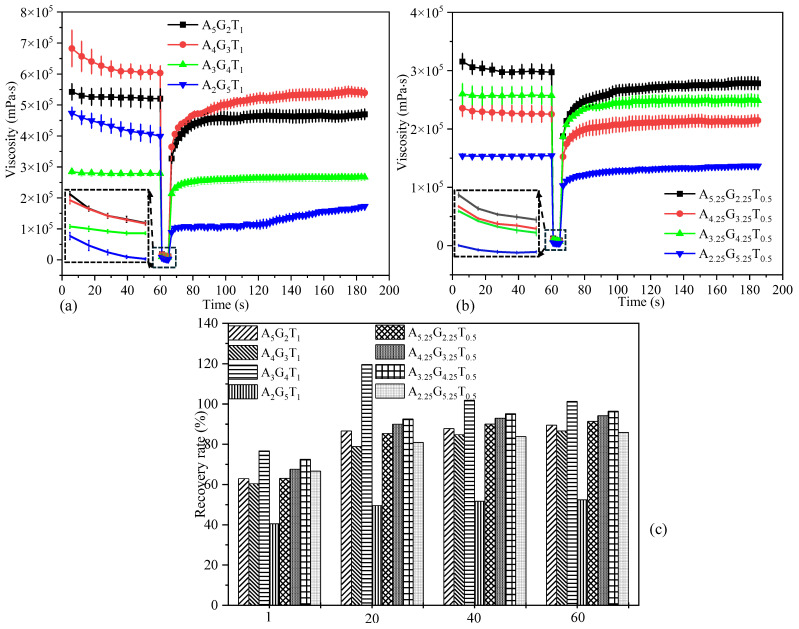
Three-point interval thixotropic test (3iTT) of various percentages of alginate and gelatin infilled with: (**a**) 1% TO-NFC, (**b**) 0.5% TO-NFC, and (**c**) recovery rate of various compositions after 1, 20, 40, and 60 s of release from the nozzle tip.

A thixotropy test with three different intervals such as at-rest, shear, and recovery was performed on all compositions to assess the recovery rate. These data are crucial prior to conducting the printing process, as they directly impact the accuracy of filament shape. The first interval represents the resting state of the sample, the second simulates the hydrogel’s structural breakdown under high shear during extrusion, and the third reflects the recovery rate, indicating the ability to restore its structure post-extrusion, as shown in Figure 3. In the first interval, a shear rate of 1.0 s^−1^ was applied for 60 s, followed by an increase to 100 s^−1^ for 5 s in the second interval. As shown in Figure 3c, the recovery rates of all eight compositions were evaluated at 2, 20, 40, and 60 s, revealing a consistent upward trend. Most compositions, except A_2_G_5_T_1_, achieved recovery rates exceeding 80% within 20 s after extrusion. This indicates that the deposited filaments are likely to retain their shape and geometric accuracy.

#### 2.1.4. Loss and Storage Modulus

The amplitude sweep test, conducted at a constant frequency of 1 Hz in this study, is employed to delineate the linear viscoelastic region (LVR) of the material prior to subsequent frequency sweep analysis. During this test, the deformation amplitude or shear stress amplitude is systematically varied while maintaining a constant frequency. The resultant complex modulus (G* = G′ *+ iG*″) comprises the storage modulus (G′, representing solid-like behavior) and loss modulus (G″, representing liquid-like behavior). At low shear strain levels, all compositions exhibited a predominance of solid-like behavior over liquid-like, which persisted until a critical point of intersection, known as the gel point. The LVR establishes the range within which testing can be performed without compromising the structural integrity of the sample, thereby preserving its deposit characteristics without inducing permanent deformation.

**Figure 4:**
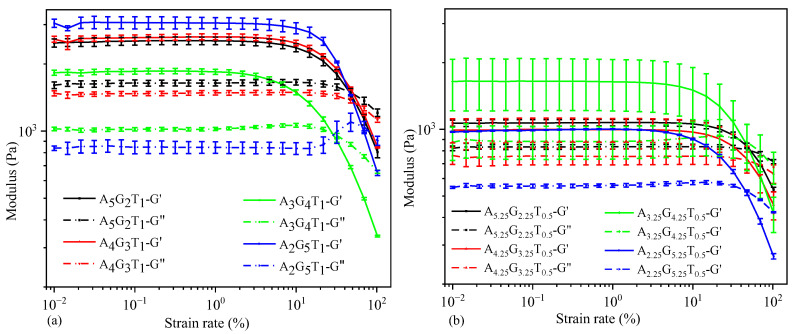
Amplitude test: loss (*G*″) and storage modulus (*G*′) of various percentages of alginate and gelatin infilled with: (**a**) 1% TO-NFC and (**b**) 0.5% TO-NFC.

Figure 4 represents the strain rate at the LVR, along with the corresponding G′ and G″ values, as identified. All compositions demonstrated a ‘gel structure’, evidenced by G′ > G″ within the LVR. Beyond the intersection point, termed the flow point, liquid-like behavior began to dominate, inducing material flow. This flow stress value provides insight into the relationship between extrusion pressure and material flow, with the effective pressure required to exceed this LVR strain rate for successful nozzle extrusion. Notably, with higher TO-NFC and gelatin percentages, both G′ and G″ showed higher values.

### 2.2. Prediction of Viscosity as a Function of Weight of Constituents and Shear Rate

#### 2.2.1. Polynomial Fit

**Figure 5:**
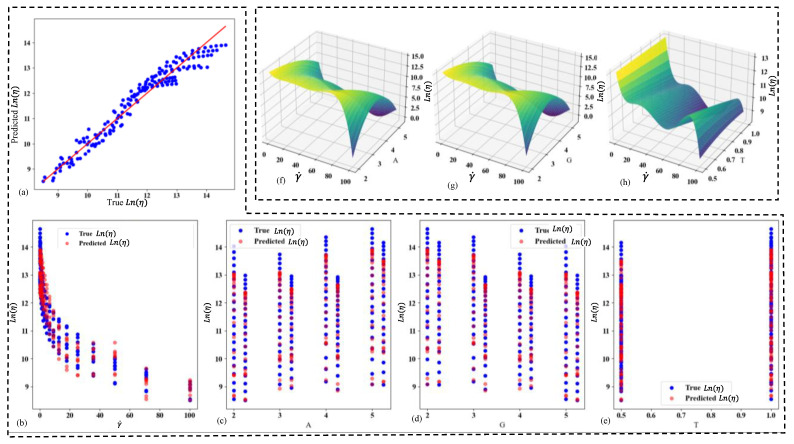
(**a**) Scatter plot of predicted vs. true viscosity in Ln scale; scatter plot of predicted vs. true viscosity with respect to (**b**) shear rate, (**c**) weight of A, (**d**) weight of G, (**e**) weight of T; viscosity distribution with the change of shear rate and (**f**) weight of A, (**g**) weight of G, (**h**) weight of T.

The polynomial fit model has been used to predict viscosity as a function of the constituents’ weights (A for alginate, G for gelatin, T for TO-NFC), and shear rate ($\dot{\gamma }$) is a fourth-degree polynomial regression. This model achieved a high coefficient of determination (R^2^ value) of 0.95, indicating that it explains 95% of the variability in the viscosity data. The mean absolute error (MAE) of 0.28 suggests a reasonably good fit, with predictions deviating from actual values by an average of 0.28 units. All coefficients for 4th order polynomial predictive model are shown in Table S1. The model’s performance is shown in Figure 5. Figure 5a shows the true vs. predicted viscosity values, demonstrating the overall accuracy of the model. The next four scattered graphs (Figure 5b–e) display the true vs. predicted viscosity with respect to each parameter ($\dot{\gamma }$, A, G, and T). These graphs help to visualize how well the model predicts viscosity for different levels of each constituent and shear rate. The final three graphs (Figure 5f–h) are surface plots showing the viscosity distribution with respect to SR and each of the constituents (A, G, and T). These 3D visualizations allow for a more comprehensive understanding of how viscosity changes with varying levels of shear rate and each constituent. The surface plots reveal that alginate (A) has a higher impact on viscosity with respect to shear rate compared to gelatin (G) and TO-NFC (T). This is evident from the steeper gradients and more pronounced curvature in the surface plot for alginate compared to those for gelatin and TO-NFC. This fourth-degree polynomial model provides a comprehensive tool for predicting bioink viscosity based on composition and shear rate, with high accuracy as indicated by the R^2^ value. The visualizations offer insights into the complex relationships between the constituents, shear rate, and resulting viscosity, which can be valuable for optimizing bioink formulations in 3D bioprinting applications.

#### 2.2.2. Decision Tree

**Figure 6:**
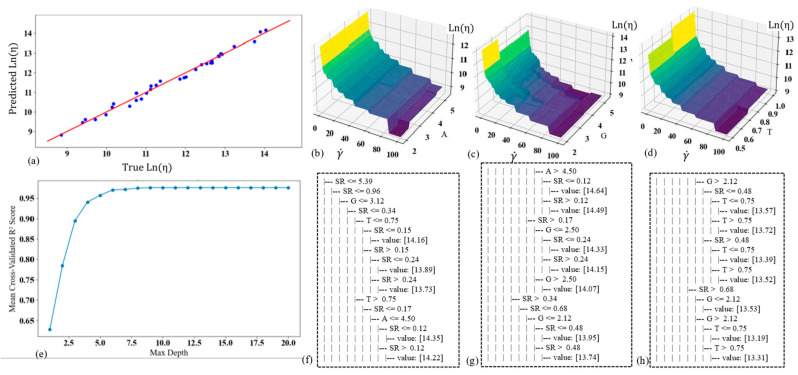
(**a**) Scatter plot of predicted vs. true viscosity in Ln scale; viscosity distribution with the change of shear rate and (**b**) weight of A, (**c**) weight of G, (**d**) weight of T; (**e**) optimum number of trees, (**f**–**h**) some sample rules extracted from the original set of rules shown in Table S2 of supplementary information.

The decision tree model is used to predict viscosity as a function of the constituents’ weights (A for alginate, G for gelatin, T for TO-NFC), and shear rate ($\dot{\gamma }$) is a hierarchical, tree-like structure that makes decision rules based on input features to predict the output. This model achieved a very high coefficient of determination (R^2^ value) of 0.988, indicating that it explains 98.8% of the variability in the viscosity data. The mean absolute error (MAE) of 0.13 suggests a good fit, with predictions deviating from actual values by an average of 0.13 units. The model’s performance and characteristics are shown in Figure 6. The first scattered graph shows the true vs. predicted viscosity values, demonstrating the overall high accuracy of the model across all data points. The close alignment of points to the diagonal line indicates strong predictive performance. Figure 6b–d graphs are surface plots showing the viscosity distribution with respect to $\dot{\gamma }$ and each of the constituents (A, G, and T). These 3D visualizations allow for a comprehensive understanding of how viscosity changes with varying levels of shear rate and each constituent, helping to identify any non-linear relationships or interactions between variables. Figure 6e illustrates determining the optimal tree depth. It shows the relationship between the maximum depth of the tree and the mean cross-validated R^2^ value. The optimal depth was found to be 9, balancing model complexity with predictive performance and avoiding overfitting. Figure 6f–h presents sample rules extracted from the original set of decision rules (detailed in Table S2 of the supplementary information). These rules provide insight into the decision-making process of the tree, showing how the model splits the data based on different feature thresholds to make predictions. The decision tree model’s high R^2^ value and relatively low MAE indicate its strong performance in predicting bioink viscosity based on composition and shear rate. The model captures complex relationships between the input variables and viscosity, providing an interpretable tool for bioink formulation and optimization.

#### 2.2.3. Random Forest

**Figure 7:**
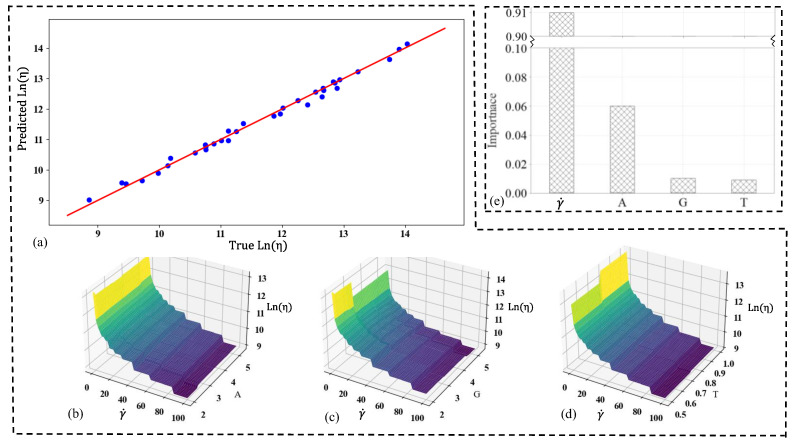
(**a**) Scatter plot of predicted vs. true viscosity in Ln scale; viscosity distribution with the change of shear rate and (**b**) weight of A, (**c**) weight of G, (**d**) weight of T, (**e**) importance factors of shear rate, and weight of A, G, and T.

The random forest model was used to predict viscosity as a function of the constituents’ weights (A for alginate, G for gelatin, T for TO-NFC) and shear rate ($\dot{\gamma }$) is an ensemble learning method that combines multiple decision trees to make predictions. This model achieved an exceptionally high coefficient of determination (R^2^ value) of 0.99, indicating that it explains 99% of the variability in the viscosity data. The MAE of 0.088 suggests a very good fit, with predictions deviating from actual values by an average of only 0.088 units. The model’s performance is shown in Figure 7. Figure 7a shows the true vs. predicted viscosity values, demonstrating the overall high accuracy of the model across all data points. The close alignment of points to the diagonal line indicates strong predictive performance. Graphs shown in Figure 7b–d are surface plots showing the viscosity distribution with respect to $\dot{\gamma }$ and each of the constituents (A, G, and T). These 3D visualizations allow for a comprehensive understanding of how viscosity changes with varying levels of shear rate and each constituent. The surface plots reveal that gelatin (G) has a higher impact on viscosity with respect to shear rate compared to alginate (A) and TO-NFC (T). This is evident from the steeper gradients and more pronounced curvature in the surface plot for gelatin compared to those for alginate and TO-NFC. This insight suggests that adjusting the gelatin content could have a more significant effect on the bioink’s viscosity response to shear rate than modifying the other constituents.

Figure 7e is an important plot, which ranks the features ($\dot{\gamma }$, A, G, T) based on their influence on the model’s predictions. This plot demonstrates that shear rate (SR) has the highest importance in controlling the viscosity, followed by the constituent weights. This finding highlights the critical role of shear rate in determining the bioink’s viscosity, which is crucial information for optimizing the bioprinting process. The random forest model’s high R^2^ value and low MAE indicates its superior performance in predicting bioink viscosity based on composition and shear rate. The model captures complex, non-linear relationships between the input variables and viscosity, providing a powerful bioink formulation and optimization tool. The RF model offers valuable insights into the relative influence of each factor, which can further guide researchers in focusing their efforts on the most impactful parameters when designing bioinks for user-specific applications.

#### 2.2.4. Optimization

Optimization was performed using differential evolution, a global optimization algorithm capable of handling non-linear and complex problems effectively. The parameter space was constrained to observed ranges in the dataset to ensure physically meaningful solutions. Two optimization scenarios were explored. Firstly, without additional shear rate constraints, the optimization yielded the following results: ($\dot{\gamma },\;A,\;G,\;T$) = (0.68, 2.81, 4.93, 0.63), and the predicted Ln (viscosity) was 12.32, which is equivalent to 224,134 mPa·s. These results fall within the target viscosity range, demonstrating the efficacy of the optimization approach in identifying parameter sets that meet the desired structural integrity criteria.

Finally, to investigate practical scenarios requiring a higher shear rate, a constraint of $\dot{\gamma }>50$ was added. Because, in the real world, 3D bioprinting processes’ shear rates at the nozzle tip often exceed 50 s^−1^ due to the high extrusion pressures required to maintain a consistent flow of bioinks [59,60], this adjustment ensures the optimized parameters are reflective of realistic printing conditions, where shear-thinning behavior and flow dynamics are critical to structural integrity. The resulting optimal parameters were ($\dot{\gamma },\;A,\;G,\;T$) = (58.03, 5.10, 2.68, 0.93), and predicted Ln(viscosity) was 9.92, which is equivalent to 20,333 mPa·s. The results indicate that while $\dot{\gamma }>50$ the constraint was satisfied, the predicted viscosity did not fall within the desired range of 11.52–13.12. This suggests that additional adjustments to input ranges or model constraints may be needed to achieve practical results under high-shear conditions.

### 2.3. Microstructure of Hybrid Hydrogels

#### 2.3.1. Scanning Electron Microscopy (SEM)

**Figure 8:**
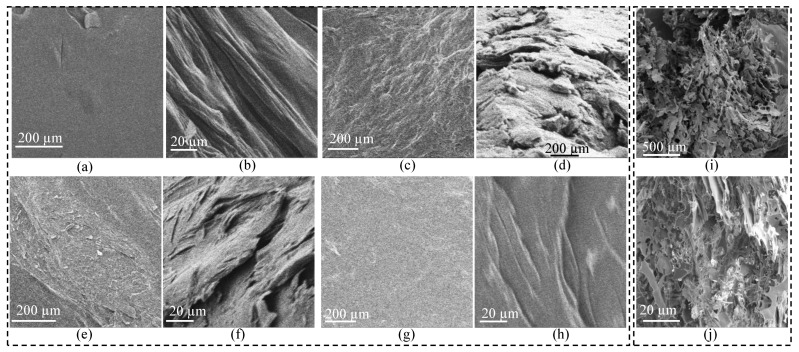
SEM images: (**a**,**b**) A_2.25_G_5.25_T_0.5_ surface and cross-section, respectively; (**c**,**d**) A_2_G_5_T_1_ surface and cross-section, respectively; (**e**,**f**) A_5.25_G_2.25_T_0.5_ surface and cross-section, respectively; and (**g**,**h**) A_5_G_2_T_1_ surface and cross-section, respectively; cross sections of freeze-dried (**i**) A_5_G_2_T_1_ and (**j**) A_2.25_G_5.25_T_0.5_. Scale for all surfaces and cross-sections are 200 µm and 20 µm, respectively.

The morphological microstructure was conducted using SEM. Imaging was performed on the surface and cross-sections of all four dried compositions such as A_2.25_G_5.25_T_0.5_, A_2_G_5_T_0.5_, A_5.25_G_2.25_T_0.5_, and A_5_G_2_T_1_ to analyze their microstructure morphologically, as shown in Figure 8a–j. A smooth cell structure and homogeneous distribution of alginate, gelatin, and TO-NFC were observed. This homogeneous distribution has resulted from the strong physical interaction between alginate, gelatin, and TO-NFC. Where most of the surface was smooth, the cross-sections showed different textures. With increasing the solid load of gelatin, higher roughness was observed. A work was reported recently where the surface and cross-section of alginate–gelatin compositions were imaged. The authors showed that, while the resulting surface was smooth, the freeze-dried pore size was bigger for the compositions with more gelatin [38]. We analyzed the microstructure with and without freeze-drying. Even though the first set of samples shown in Figure 8a–h were not freeze-dried, they showed similar patterns: smooth surface and rough cross-section with increasing the solid load of gelatin. To analyze the impact of gelatin compared to alginate, two samples with the highest and lowest amount of gelatin such as A_2.25_ G_2.25_T_0.5_ and A_5_G_2_T_1_ were chosen for freeze-dried SEM analysis. Samples were freeze-dried to remove water while preserving structural integrity and porosity, thus enabling imaging via SEM. This is crucial to understand as the resulting morphology determines the mechanical properties of the scaffold and the efficiency of cell infiltration and nutrient transport [61,62]. This process also ensures minimal shrinkage and deformation, making it ideal for tissue engineering applications. Figure 8i,j represents a similar pattern as reported in [38]. In another work, it was reported that, with a rough surface of alginate and gelatine composition, encapsulated stem cells can have better anchoring sites to spread and connect [63]. Therefore, systematic control of alginate and gelatin can help improve the cell viability of the prepared bioink after printing them.

#### 2.3.2. Fourier Transformation Infrared (FTIR)

**Figure 9:**
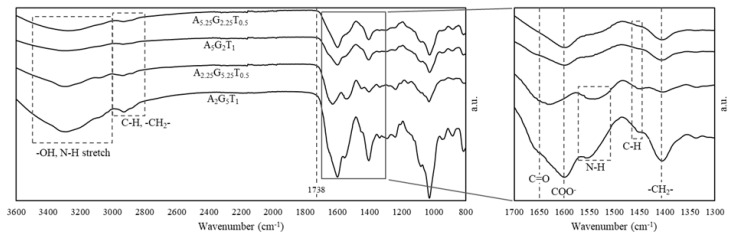
FTIR spectra of select formulations.

FTIR spectra were collected on A_5.25_G_2.25_T_0.5_, A_5_G_2_T_1_, A_2.25_G_5.25_T_0.5_, and A_2_G_5_T_1_. The analysis shown in Figure 9 provides insight into the chemical structure of these formulations. Immediately, the lack of an aldehyde peak at 1738 cm^−1^ indicates that crosslinking has not occurred between alginate and gelatin for these formulations [39], as expected. Broad peaks from 3000 to 3500 cm^−1^ are characteristic of both O-H and N-H stretching [64] in alginate and gelatin, respectively. Peaks centered at 2900 cm^−1^ can be attributed to elemental functional groups found in lignocellulosic fibers [65], primarily C-H and -CH_2_- groups [66], with minor contributions from NH groups [67]. Moreover, peaks boxed at 2900 and 1449 cm^−1^ are attributed to the hydrocarbon content of both alginate and gelatin [66,68]. The shift in the position of Amide I bands (1650 cm^−1^) shown in high gelatin content formulations (A_2_G_5_T_1_, A_2.25_G_5.25_T_0.5_) is associated with backbone conformation and hydrogen bonding pattern [64]. The asymmetric COO^−^ stretching vibration (1600cm^−1^) of A_2_G_5_T_1_, A_5_G_2_T_1_, and A_5.25_G_2.25_T_0.5_ formulations are associated with polysaccharide content [66]. Interestingly, A_2.25_G_5.25_T_0.5_ does not exhibit a peak in this location and may indicate that this functional group exhibits a different interaction between components as compared to the other samples, though more work is required to elucidate these differences. High alginate content formulations (A_5_G_2_T_1_, A_5.25_G_2.25_T_0.5_) do not exhibit the same Amide II (1510–1580 cm^−1^) band [64] as that of low alginate content (A_2_G_5_T_1_, A_2.25_G_5.25_T_0.5_). This is likely due to the relative increase in NH and CN groups of gelatin [66].

### 2.4. 3D Bioprinting

**Figure 10:**
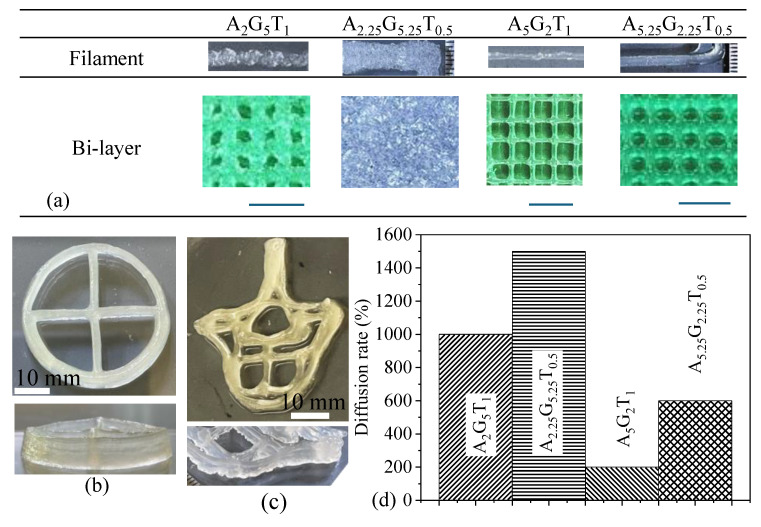
(**a**) Filaments and bilayer fabricated using compositions mentioned, (**b**,**c**) regular and freeform constructs fabricated with A_5_G_2_T_1_. (**d**) Diffusion rate of the filament with respect to the 0.41 mm nozzle.

To assess the printability, a set of four compositions such as A_2_G_5_T_1_, A_2.25_G_5.25_T_0.5_, A_5_G_2_T_1_, and A_5.25_G_2.25_T_0.5_ were extruded through a 0.41mm dispensing nozzle using 140 kPa air pressure, 5 mm/s print speed, and 0.15 mm Z-height. Notably, filaments with a lower ratio of (Gelatin Weight)/(Alginate×TONFC Weight) demonstrated better size and shape characteristics. The (Gelatin Weight)/(Alginate×TONFC Weight) ratios for A_2_G_5_T_1_, A_2.25_G_5.25_T_0.5_, A_5_G_2_T_1_, and A_5.25_G_2.25_T_0.5_ were 2.5, 4.66, 0.4, and 0.85, respectively. A_5_G_2_T_1_ and A_2.25_G_5.25_T_0.5_ showed the lowest and highest values, respectively, which corresponded to their filament sizes as illustrated in Figure 10a. The diffusion rate, calculated as the percentage change in filament width relative to the nozzle diameter, is depicted in Figure 10b for all filaments. As anticipated, the pore size and geometry formed by a bi-layer deposition of A_5_G_2_T_1_ were superior to other compositions, as shown in Figure 10a. Although A_5.25_G_2.25_T_0.5_ formed a defined pore, its shape became circular due to diffusion. The other two compositions, A_2_G_5_T_1_ and A_2.25_G_5.25_T_0.5_, barely created pores due to larger filament size and irregular geometry. Compositions with higher gelatin (temperature sensitive) percentages did not flow smoothly, leading to nozzle clogging and requiring higher applied pressure for continuous filament extrusion. Therefore, A_2_G_5_T_1_ and A_2.25_G_5.25_T_0.5_ were not extruded with lower applied pressure than 140 kPa to achieve acceptable filament size and shape. The elevated temperature range from 30 to 55 °C resulted in a smooth filament diameter. However, since cell-laden bioink printed at temperatures above 37 °C can compromise cell viability, we excluded bioink with higher gelation ratio as a candidate for encapsulating cells. Since A_5_G_2_T_1_ showed a better chance to create filament with better size and shape, we used this material composition to fabricate a scaffold having a free-form shape with 15 layers and 5% infill as shown in Figure 10b,c. The scaffold maintained its geometry and build height.

### 2.5. Biocompatibility

**Figure 11:**
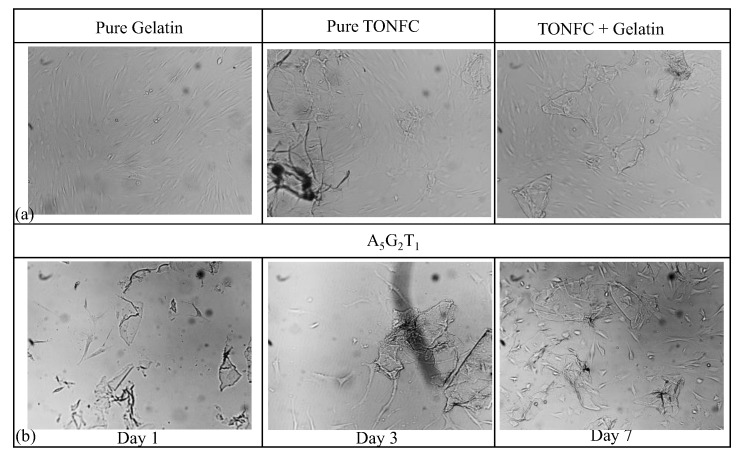
Biocompatibility tests: 2D hMSC cell culture with 7 incubation days and with the presence of (**a**) pure gelatin, TONFC, and a mix of gelatine and TONFC; (**b**) A_5_G_2_T_1_ at incubation days 1, 3, and 7.

hMSCs were mixed with pure gelatin, pure TONFC, and mixes of TONFC and gelatin. Mixes of constituents were incubated, and imaged at days 1, 4, and 7, respectively. It was observed that the number of cells increased with time. Figure 11a shows only the cell viability data for 7 days. Finally, A_5_G_2_T_1_ composition was also mixed with hMSCs, incubated, and imaged at days 1, 4, and 7, respectively. Figure 11b shows that cells expressed their morphology with the presence of A_5_G_2_T_1_ by anchoring and spreading on the petri dish surface, meaning that the composition is biocompatible. It is also observed that the number of cells increased with the increase of the incubation time from day 1 to day 7. This is another indication that cells may grow with the presence of A_5_G_2_T_1_. In the future, we plan to 3D bioprint scaffold with this bioink and test cell viability, proliferation, and migration to targeted tissue.

### 2.6. Discussion

The 3D bioprinting community often recommends increasing solid content to improve the shape fidelity of bioprinted scaffolds, though this can hinder the cell viability [69,70,71]. Understanding how specific constituents and shear rates influence viscosity can aid in tailoring bioinks to achieve the desired balance between shape fidelity and cell viability. This insight allows the preparation of hybrid hydrogels with varying solid contents that maintain comparable viscosities [25,72]. Conversely, different compositions with the same solid content can produce significantly different viscosities. Given the shear-thinning behavior of hydrogels commonly used in 3D bioprinting, achieving the necessary viscosity for smooth extrusion can be controlled by adjusting the shear rate, which depends on factors such as applied pressure and nozzle diameter. As a result, similar viscosities can be obtained over a wide range of shear rates by optimizing material composition and printing parameters. This paper represents a framework that can be followed to develop any potential hybrid hydrogels for the extrusion-based bioprinting process. The predictive model of viscosity in terms of constituents’ weight and shear rate can reduce the exhaustive experiments and assist in finding an effective combination of materials and process parameters to fabricate constructs with defined architecture and ensure better cellular activities.

In this study, three different ML algorithms were employed to predict the viscosity of a series of novel hybrid hydrogels composed of alginate, gelatin, TO-NFC, and considering shear rate: a polynomial fit model, a decision tree model, and a random forest model. Each model’s performance was evaluated using the coefficient of determination (R^2^) and mean absolute error (MAE). The comparison of R^2^ value and MAE is shown in Table 1. The polynomial fit model achieved an R^2^ value of 0.95 and an MAE of 0.28, indicating good overall performance but with room for improvement. The decision tree model showed enhanced predictive capability with an R^2^ value of 0.988 and an MAE of 0.13, demonstrating better accuracy and lower error compared to the polynomial fit. However, the random forest model emerged as the top performer, boasting the highest R^2^ value of 0.99 and the lowest MAE of 0.09. Comparison graphs were presented to visualize the performance of each model. These included scatter plots of predicted versus actual viscosity values (Figure 5a, Figure 6a and Figure 7a), which clearly illustrated the superior fit of the random forest model, with data points clustering more tightly around the ideal prediction line. Surface plots for each model demonstrated how they captured the relationships between viscosity, shear rate, and individual hydrogel components, with the random forest model showing the most nuanced and accurate representations.

The results of this study align with previous research demonstrating the effectiveness of Random Forest models in capturing complex, non-linear relationships in rheological data, outperforming simpler models like polynomial fit regression and decision tree [73]. Similar findings have been reported for predictive frameworks in bioink and polymeric systems, where random forest consistently achieves superior accuracy and reliability in viscosity modeling under varying shear rates [74,75].

Based on these results, we recommend the random forest model as the best choice for users aiming to reduce overall experimentation in future 3D bioprinting applications. Its superior R^2^ value and lowest MAE indicates that it provides the most accurate and reliable predictions of hydrogel viscosity across various compositions and shear rates. By employing this model, researchers can more efficiently explore the vast parameter space of hydrogel compositions, potentially reducing the number of physical experiments required to optimize bioink formulations. This approach can significantly streamline the bioink development process, saving time and resources while potentially leading to more optimized and consistent 3D bioprinting outcomes.

During 3D printing, we observed the direct effect of gelatin as a form of ‘gelatin/(alginate × TONFC)’ on the filament shape fidelity that can drive users to select the right ratio of bioink constituents to print defined filament width and consequently the pore geometry and 3D construct. Reportedly, gelatin has a better effect on cellular activities; therefore, the predictive model can help fine-tune the viscosity of a bioink to achieve printed construct with defined architecture and higher cell viability. Microstructural analysis using SEM supports this claim. Two examples of printed construct indicate the capability to fabricate large-scale (cm-scale) constructs using one of the hybrid hydrogels, e.g., A_5_G_2_T_1_. Finally, the growth trend of hMSCs mixing with A_5_G_2_T_1_ in various incubation days shows a great prospect for these hydrogels to be used as potential bioinks for extrusion-based 3D bioprinting purposes.

## 3. Conclusions

This study presents a framework for developing hybrid hydrogels for extrusion-based bioprinting. Various ML algorithms were compared by performance to predict viscosity in the context of constituents’ weight and shear rate. After comparing polynomial fit, decision tree, and random forest models, we found the random forest model to be superior, with the highest R^2^ (0.99) and lowest MAE (0.09). This approach significantly reduces the need for exhaustive experiments in bioink development. The random forest model’s accuracy in predicting hydrogel viscosity based on constituents’ weight and shear rate allows researchers to efficiently explore a wide range of parameters, potentially streamlining the optimization of bioink formulations. This framework is adaptable to various hybrid hydrogels and bioprinting applications, enabling the systematic development of constructs with defined architectures and improved cellular activities. In conclusion, this study advances the rational design of bioinks for 3D bioprinting, combining experimental data with machine learning to create a powerful predictive tool. In this study, the ML models were developed using rheological data generated from 169 rheometer sweeps across varying shear rates and bioink compositions. For practitioners in the 3D bioprinting community, this approach demonstrates that with sufficient rheological measurements as base input, the model can accurately predict viscosities and guide the optimization of bioink formulations. This ensures accessibility for professionals, such as cellular biologists, who may not have advanced expertise in ML but seek practical tools for bioprinting applications. Therefore, this approach has the potential to accelerate innovation in tissue engineering and regenerative medicine, facilitating the efficient development of complex, functional tissue constructs.

## 4. Materials and Methods

### 4.1. Hybrid Hydrogel Preparation

**Figure 12:**
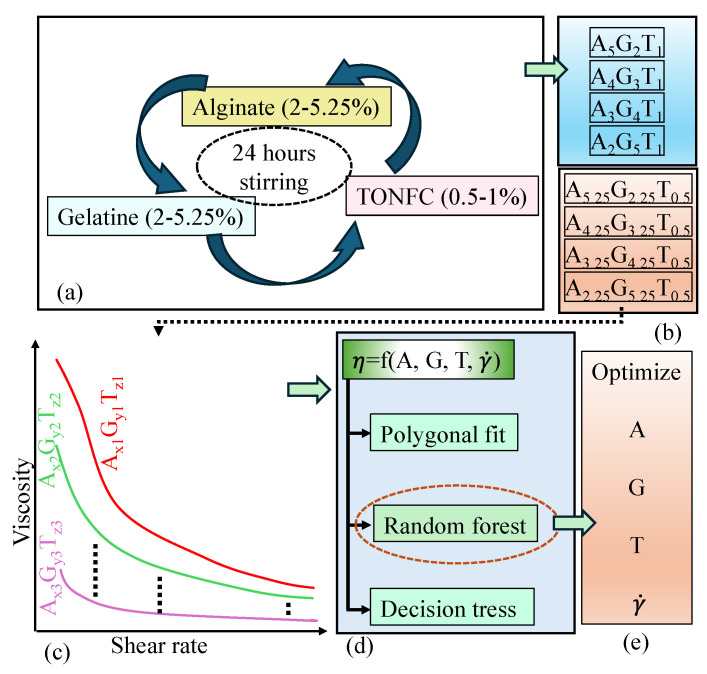
(**a**–**e**) An overview of the overall framework that starts with the material preparation followed by rheological data collection and the use of various ML algorithms to predict viscosity.

Dry TEMPO nano-fibrillated cellulose (TO-NFC) [(C_6_H_10_O_5_)_x_(C_6_H_9_O_4_CO_2_Na)_y_] with a carboxylate content ranging from 0.2 to 2 mmol/g solids was obtained from the Process Development Center (PDC) at the University of Maine. Medium-viscosity alginate (A) (viscosity ≥ 2000 cps in a 2% aqueous solution) and gelatin (G) (gel strength ~300 g Bloom, 100–200 μg/cm^2^) were sourced from Sigma–Aldrich (St. Louis, MO, USA). These were combined with TO-NFC following the protocol outlined in Figure 12. A total of eight compositions were created, varying the proportions of A, G, and T to evaluate the influence of each component on the overall composition. To reflect the impact of each element in the compositions, the followings were chosen: 2, 2.25, 3, 3.25, 4, 4.25, 5, 5.25% alginate; 2, 2.25, 3, 3.25, 4, 4.25, 5, 5.25% gelatin; and 0.5 and 1% TO-NFC. These were mixed systematically to prepare eight compositions such as A_5.25_G_2.25_T_0.5_, A_5_G_2_T_1_, A_4.25_G_3.25_T_0.5_, A_4_G_3_T_1_, A_3.25_G_4.25_T_0.5_, A_3_G_4_T_1_, A_2.25_G_5.25_T_0.5_, and A_2_G_5_T_1_, maintaining a total solid content of 8%. All numerical subscripts represent the solid load of the component mixed into the water to prepare the material compositions.

### 4.2. Rheological Properties

Rheological properties such as viscosity, shear stress, shear thinning behavior, linear viscoelastic range, and recovery rate were assessed using a rotational rheometer (MCR 102, Anton Paar, Graz, Austria), employing a parallel plate configuration with a flat plate diameter of 25.0 mm. The distance between the plates was set to 1.0 mm. Usually, the gelatin used for 3D bioprinting application was processed with a range of 40–70 °C [76,77,78]. We collected our experiment at 55 °C temperature in anticipation of conducting the extrusion process under similar conditions. To assess rheological properties, various rheological analyses were executed, such as (i) flow curve analysis to determine shear thinning behavior, (ii) three-point interval thixotropic test (3iTT) to identify recovery rate, and (iii) amplitude test to find the gelation points. The process parameters used for rheological tests are listed in Table 3.

To assess the shear-thinning factors such as infinite and zero shear viscosity, time constant, and transition control factors of the studied compositions, the Cross model (Equation (1)) fitted to the flow curve plotted against the shear strain rate [26].

(1) $$\eta =\eta_{\infty }+\frac{\eta_{0}-\eta_{\infty }}{1+{\left(t\dot{\gamma }\right)}^{m}}$$

where $\eta_{\infty }$, $\eta_{0}$, *t*, and *m* are viscosity at infinite shear, viscosity at zero-shear, time constant, and transition control factor, respectively.

The Herschel–Bulkley model (Equation (2)) [34,35] was fitted to the shear stress versus shear rate curves to determine the consistency index and flow index:

(2) $$\tau =\tau_{0}+k{\dot{\gamma }}^{n}$$

where $\tau_{0}$, *k*, and *n* are yield stress, consistency index, and flow index, respectively. Once *k* and *n* are estimated, the shear stress at different shear strains can be determined using Equation (2).

### 4.3. Microstructure and Chemical Bonding

#### 4.3.1. Scanning Electron Microscopy and Freeze Drying

The select samples were freeze-dried in a Labconco 120mL Complete Fast-Freeze Flask using a Labconco FreezeZone 1L Freeze Dry System (Labconco Corporation, Kansas City, MO, USA); these materials were frozen for 72 h at −80 °C and then dried at 0.024 mBar and −47 °C for 24 h prior to imaging. Imaging was focused on the surface and cross-sections of samples, which were mounted on 0.5-diameter pin-type stubs. Samples were coated with approximately 15 nm of Au/Pd using an SPI Supplies SPI-MODULE Sputter Coater. Imaging of the samples was carried out with a TescanMira3field Emission SEM (Brno, Czech Republic) operating at 10 kV with a Secondary Electron (SE) detector. Working distance (WD) for imaging ranged between 30 and 35 mm with magnifications of 200×–1900× scale.

#### 4.3.2. Scanning Electron Microscopy and Freeze Drying

A PerkinElmer Frontier FTIR (Waltham, MA, USA) with Attenuated Total Reflectance (ATR) attachment (Diamond/ZnSeGe crystal) was utilized in the 650–4000cm^−1^ range, averaging 4 scans per experiment. Data were collected on unreacted liquid formulations with UATR 3.0mm Sample Position Plate (Part No. L1202023).

### 4.4. 3D Printing and Characterization

Utilizing an extrusion-based 3D bioprinter (BioX, CELLINK, Boston, MA, USA), filaments and scaffolds were fabricated. Hybrid hydrogels were prepared as described in Section 4.1, loaded into a 3.0 mL disposable nozzle, and extruded pneumatically onto a stationary build platform. The nozzle and bed temperatures of the 3D bioprinter were adjusted based on the amount of gelatin in the composition and ranged from 30 to 55 °C. However, when bioprinting with cell-laden bioink, the nozzle temperature is expected to be constrained to 37 °C to ensure maximum cell viability. In such cases, we plan to adjust the printing process parameters as outlined in our earlier publications to ensure the accuracy of filament and scaffold geometries [79,80]. The design and vectorized toolpath of a scaffold were created using Rhino 6.0 (https://www.rhino3d.com, accessed on 16 June 2024) (Robert McNeel and Associates, Seattle, WA, USA), a Visual Basic-based Computer-Aided Design (CAD) software. Slicer (https://www.slicer.org, V: 1.3.0, accessed on 16 June 2024), a G-code generator software, was used to create a Bio-X compatible file containing toolpath coordinates and process parameters. The bioprinting process followed the fashion of layer-by-layer material deposition. Three filaments were produced for each measurement. Captured under the CK Olympus bright field microscope (Shinjuku, Tokyo, Japan), images of the fabricated filaments were taken within 1–2 min of printing, minimizing exposure time. Filament width was determined using Image J (developed by the National Institute of Health).

#### Diffusion Rate and Printability

The filament that has been extruded should display a distinct structure with an even surface and consistent thickness, enabling the formation of uniform grids and square pores. However, due to the liquid-like state of the hydrogel, the filament can spread and create a circular pore. The rate of change of filament width (*FW*) compared to the nozzle diameter (*ND*), e.g., diffusion rate (*DR*) [11], was determined using the following equation:

(3) $$DR=\frac{FW}{ND}\times 100\%$$

The solid-like state of the hydrogel can require higher applied pressure to extrude material resulting in irregular pore geometry. The circularity (*C*) of an enclosed area is defined using the following equation:

(4) $$C=\frac{4\pi A_{a}}{L^{2}}$$

where *L* and *A_a_* are the perimeter and the actual area of the enclosed area, respectively. The circularity is 1 for a circle, while it is *π*/4 for a square shape. The printability of hydrogel (*P_r_*) [81] is defined using the following equation:

(5) $$P_{r}=\frac{\pi }{4}\frac{1}{C}=\frac{L^{2}}{16A_{a}}$$

A *P_r_* value lower than 1 signifies a circular pore structure, while a value exceeding 1 indicates a non-uniform pore arrangement. The targeted *P_r_* value is 1, indicating a ’square’ configuration.

### 4.5. Machine Learning Algorithms

#### 4.5.1. Polynomial Fit

To model the complex relationship between viscosity and the constituent weight of hybrid hydrogels, as well as the shear rate, a polynomial regression approach has been employed. A set of 169 data was used for this analysis. The viscosity (η) was expressed as a function of the weight percentages of alginate (A), gelatin (G), TONFC (T), and shear rate ($\dot{\gamma }$). The governing equation of an *nth*-order polynomial model for the viscosity can be expressed as:

(6) $$\eta =\sum_{i=0}^{n}\sum_{j=0}^{n-i}\sum_{k=0}^{n-i-j}\sum_{l=0}^{n-i-j-k}\beta_{ijkl}A^{i}G^{j}T^{k}{\dot{\gamma }}^{l}$$

where $\beta_{ijkl}$ are the coefficients of the polynomial, and the indices *i*, *j*, *k*, and *l* range from *0* to *n*, such that i+j+k≤n. This equation accounts for all possible combinations of the variables up to the *nth* power, capturing the intricate dependencies and interactions among the weight of alginate, gelatin, TONFC, and shear rate with viscosity. In this work, a fourth-order model is considered and compared it with higher-order models. The Akaike Information Criterion (AIC) has been used to balance model complexity and goodness of fit. The polynomial fit was validated by comparing predicted values against experimental data, with the mean absolute error (MAE) used as a metric for model performance measure. The robustness of the model was further assessed through cross-validation, a randomly sampled 80% (135) of the data for training and the rest 20% (34) of the data for testing the model over 100 iterations. This polynomial regression approach provides a comprehensive framework for predicting bioink viscosity across a range of compositions and shear rates, facilitating the optimization of 3D bioprinting parameters.

#### 4.5.2. Decision Tree

In this study, a decision tree-based model has been adopted to predict the viscosity (η) of hybrid hydrogels based on the weight percentages of alginate (A), gelatin (G), TONFC (T), and shear rate ($\dot{\gamma }$). Decision trees are powerful tools for regression analysis, capable of capturing non-linear relationships between variables [82]. The decision tree algorithm recursively partitions the data into subsets based on the feature that provides the highest information gain, resulting in a tree structure where each node represents a decision result based on the input variables and decision rules [83]. The governing equation for the decision tree model can be represented as a piecewise function, where the viscosity (η) is predicted based on the decision rule defined at each node:

(7) $$\eta =f_{i}\left(A,\;G,\;T,\dot{\gamma }\right)=\left\{\;\begin{array}{c} \eta_{1}\;for\;1^{st}\;decision\;rule\;\\ \eta_{2}\;for\;2^{nd}\;decision\;rule\\ \ldots \ldots \ldots \ldots \ldots \ldots \ldots \;\\ \eta_{d}\;for\;d^{th}\;decision\;rule \end{array}\right.$$

Each $f_{i}$ represents a regression function applied to a specific subset of the data, defined by the decision rule-based at the nodes leading to that subset. To manage the high variance and overfitting issue, the decision tree structure has been optimized using the pruning tree strategies. A balanced subtree structure has been obtained so that it minimizes the test error using cross-validation and cost complexity approach. The case study results demonstrated that the decision tree model effectively captured the complex interactions between the components and shear rate, providing accurate predictions of viscosity across a wide range of compositions and conditions. This approach offers a valuable tool for optimizing bioinkformulations in 3D bioprinting applications.

#### 4.5.3. Random Forest

In this study, a random forest-based regression model has been utilized to predict the viscosity of bioinks based on the weight percentages of A, G, T, and ($\dot{\gamma }$). Random forest, an ensemble learning method, constructs multiple decision trees during model training using random subset of data and averaging outputs estimation of the individual trees, thereby enhancing predictive accuracy and robustness. The governing equation for the random forest model can be represented as:

(8) $$\eta =\frac{1}{N}\sum_{i=1}^{N}T_{i}\left(A,G,T,\dot{\gamma }\right)=\left\{\;\begin{array}{c} {\bar{\eta }}_{1}\;for\;{average\;1}^{st}\;decision\;rule\;\\ {\bar{\eta }}_{2}\;for\;average\;2^{nd}\;decision\;rule\\ \ldots \ldots \ldots \ldots \ldots \ldots \ldots \;\\ {\bar{\eta }}_{d}\;for\;average\;d^{th}\;decision\;rule \end{array}\right.$$

where *η* is the predicted viscosity, *N* is the number of trees in the forest, and $T_{i}$ represents the prediction from the *i^th^* decision subtree. The trees in the random forest grow deep and are not pruned. Thus, they have high variance in estimation with lower bias. Large sample averaging reduces the estimate variations at the end. The random forest model was validated using cross-validation techniques, demonstrating its superior performance in predicting viscosity compared to traditional regression methods. This model provides a reliable tool for optimizing bioinkformulations, ensuring precise control over the viscosity for various 3D bioprinting applications.

### 4.6. Optimization

Our previous research established that hybrid hydrogels with a viscosity range of 100–500 Pa·s can maintain defined structural integrity [24,35,84]. Based on this finding, we will optimize the concentrations of alginate, gelatin, TO-NFC, and the shear rate within this viscosity range using the predictive model results. The optimization was performed using a global optimization algorithm, differential evolution, which is well-suited for non-linear and complex problems [85]. Differential evolution iteratively explores the parameter space to find the optimal solution while handling constraints through the penalty mechanism. To identify the optimal values of $\dot{\gamma }$, A, G, and T for achieving the target viscosity, we defined an objective function, $f\left(A,G,T,\dot{\gamma }\right)$ that minimizes the deviation of predicted viscosity ($\eta_{pred}$) from the desired range of 100–500 Pa·s (e.g, logarithmic transformation: 11.52–13.12). The midpoint ($\eta_{mid})$ is 12.32. Each parameter was constrained to its observed range in the dataset (e.g., $\dot{\gamma }\epsilon \left[{\dot{\gamma }}_{min},{\dot{\gamma }}_{max}\right],A\epsilon \left[A_{min},A_{max}\right],\;G\epsilon \left[G_{min},G_{max}\right],\;T\epsilon \left[T_{min},T_{max}\right]$) to ensure physically meaningful solutions. The objective function is defined as:

(9) $$f\left(\dot{\gamma },\;A,\;G,\;T\right)={\left(\eta_{pred}\left(\dot{\gamma },\;A,\;G,\;T\right)-\eta_{mid}\right)}^{2}$$

where $\eta_{pred}$ is the viscosity predicted by the trained random forest model given the parameters $\dot{\gamma },A,G,T$. The objective function focuses on reducing the deviation from this central value while ensuring that viscosity predictions remain within the acceptable range. To ensure the optimization adhered to the target range, a penalty function was introduced for cases where the predicted viscosity fell outside of the 11.52–13.12 range. The penalty was defined as:

(10) $$Penalty\left(\dot{\gamma },\;A,\;G,\;T\right)=\left\{\begin{array}{cc} \left|\eta_{pred}-11.52\right|, & if\;\eta_{pred}<11.52\;\\ \left|\eta_{pred}-13.12\right|, & if\;\eta_{pred}>13.12\\ 0, & otherwise \end{array}\right.$$

The penalty term ensured that the optimization process discouraged solutions with predicted viscosity values outside the desired range.

(11) $$F\left(\dot{\gamma },\;A,\;G,\;T\right)=f\left(\dot{\gamma },\;A,\;G,\;T\right)+Penalty\;\left(\dot{\gamma },\;A,\;G,\;T\right)$$

The penalty term ensured that the optimization process discouraged solutions with predicted viscosity values outside the desired range. If the optimized values are not practical for real-world 3D bioprinting applications, we can introduce additional constraints to ensure the results are more realistic and applicable. One such scenario is if we want to achieve $\dot{\gamma }>50$ during printing, the updated range in the dataset will be $\dot{\gamma }\epsilon \left[50,{\dot{\gamma }}_{max}\right],A\epsilon \left[A_{min},A_{max}\right],\;G\epsilon \left[G_{min},G_{max}\right],\;T\epsilon \left[T_{min},T_{max}\right],$ and the penalty function will be:

(12) $$Penalty\left(\dot{\gamma },\;A,\;G,\;T\right)=\left\{\begin{array}{c} {\left(50-\dot{\gamma }\right)}^{2}\;if\;\dot{\gamma }\leq 50\\ \begin{array}{cc} \left|\eta_{pred}-11.52\right|, & if\;\eta_{pred}<11.52\;\\ \left|\eta_{pred}-13.12\right|, & if\;\eta_{pred}>13.12\\ 0, & otherwise \end{array} \end{array}\right.$$

We observed in most of our earlier investigations that the effective shear rate during extrusion showed more than 50 s^−1^ [24,80]. This scenerio was also supported by other researchers [86].

### 4.7. Biocompatibility

Normal Human Adipose-Derived Mesenchymal Stem Cells (hMSCs) (ATCC, Manassas, Virginia) were cultured and maintained in a low-serum growth medium designed for adipose and umbilical-derived MSCs. The medium contained 2% FBS, 5 ng/mL rh FGF-basic, 5 ng/mL rh FGF-acidic, 5 ng/mL rh EGF, 2.4 mM L-Alanyl-L-Glutamine, and 0.5 mL of Penicillin–Streptomycin–Amphotericin B Solution (ATCC, Manassas, Virginia). Cells were incubated at 37 °C with 5% CO_2_, and the culture medium was refreshed twice a week. Cells at passage 3 were utilized for encapsulation, and bright-field microscopy images were obtained using a CK Olympus microscope (Shinjuku, Tokyo, Japan).

## Figures and Tables

**Table 1:** Comparative analysis of machine learning models.

Model Criteria	Polynomial Fit	Decision Tree	Random Forest
**Features**	Use a straightforward polynomial math equation	Use the recursive binary splitting algorithm	Use the bootstrap resampling algorithm
	Considers the higher-order terms and interdependencies of variables	Capture the non-linear relationships of variables	Utilize an ensemble approach to improve model performance
**Advantages**	Comparative lower but acceptable model performance with R^2^ 0.95, MAE 0.28	Comparative mid-model performance with R^2^ 0.988, MAE 0.13	Comparatively highest model performance with R^2^ 0.99, MAE 0.088
	Results in a simpler output vs. predictor model equation	Highly visual with simple decision rule-based outcome	A highly accurate model with lower biases
**Challenges**	Physical interpretation of higher-order terms may not be feasible	Higher variance and biases on the predicted model	Model interpretability and visuals are compromised
	Lower extrapolatory properties make poor model performance with newer dataset	Highly sensitive and non-robust model especially when lacking the non-linear relationship among variables	Comparatively, it requires more resources and slower processing of data

**Table 2:** Fitting Cross and Herschel model for various compositions of various percentages of alginate and gelatin infilled with T_1_ and T_0.5_. Each raw datum was used from Figure 1 and fitted accordingly as shown in Figure 2 to determine the rheological factors.

	Cross model					Herschel model			
	$\eta_{\infty }$(mPa·s)	$\eta_{0}$(mPa·s)	t (s)	m	$Adj{-R}^{2}$	$\tau_{0}$ (Pa)	K (mPa·s*^n^*)	n	$Adj{-R}^{2}$
$A_{5}G_{2}T_{1}$	6191	4,182,705	8.05	0.83	0.99	255.89	582.6	0.22	0.94
$A_{4}G_{3}T_{1}$	6908	3,476,364	10.41	0.76	0.99	200.51	570.86	0.27	0.95
$A_{3}G_{4}T_{1}$	1422	2,306,424	17.52	0.71	0.99	70.04	298.76	0.28	0.97
$A_{2}G_{5}T_{1}$	966	1,956,326	27.9	0.63	0.99	51.62	253.21	0.18	0.98
$A_{5.25}G_{2.25}T_{0.5}$	9157	2,497,101	7.06	0.75	0.99	159.78	442.08	0.26	0.95
$A_{4.25}G_{3.25}T_{0.5}$	2784	606,959	3.15	0.67	0.99	69.54	239.52	0.35	0.96
$A_{3.25}G_{4.25}T_{0.5}$	1298	693,471	5.21	0.66	0.99	77.81	228.12	0.32	0.94
$A_{2.25}G_{5.25}T_{0.5}$	1460	939,531	12.79	0.68	0.99	64.68	171.27	0.26	0.94

**Table 3:** Parameters of rheological tests for the proposed work.

Process Parameters for Rheological Tests		
Share rate (s^−1^)	Time (s)/share rate (s^−1^)	Share strain (%)
0.1 to 100	0–60/1, 61–65/100, 66–185/1	0.1 to 100

## Data Availability

The original contributions presented in this study are included in the article/supplementary material. Further inquiries can be directed to the corresponding authors.

## Supplementary Materials

The following supporting information can be downloaded at: https://www.mdpi.com/article/10.3390/gels11010045/s1, Table S1: Coefficients of all variables for 4th order polynomial predictive model; Table S2: All rules for decision tree-based model.
