# Supplementary material for: Characterization and Machine Learning-Driven Property Prediction of a Novel Hybrid Hydrogel Bioink Considering Extrusion-Based 3D Bioprinting
Source: Gels. 2025 Jan 7;11(1):45. doi: 10.3390/gels11010045 (PMC11765179; doi:10.3390/gels11010045)
Supplement: Supplementary file 1 [file gels-11-00045-s001.zip › gels-3382610-supplementary.pdf]

Supplemental Information

4th order polynomial equation to predict the viscosity for various A, G, T, and $\dot{\gamma}$

$$
\begin{aligned}
\eta &= 16.6943 - 0.0001\dot{\gamma} + 0.0009A - 0.0009G + 0.0001 * T - 0.0003\dot{\gamma}A \\
&- 0.0004\dot{\gamma}G - 0.0004\dot{\gamma}T + 0.0037A^{2} - 0.0000A\,G + 0.0032A\,T + -0.0045G^{2} + -0.0028G\,T + 0.0001T^{2} + 0.0001\dot{\gamma}^{2} \\
&+ 0.0001\dot{\gamma}^{2}G\; 0.0001\dot{\gamma}^{2}T - 0.0005\dot{\gamma}A^{2} - 0.0009\dot{\gamma}A\,G - 0.0012\dot{\gamma}A\,T - 0.0006SR\,G^{2} - 0.0014SR\,G\,T \\
&- 0.0005\dot{\gamma}T^{2} - 0.0104A^{3} + 0.0210A^{2}G + 0.0193A^{2}T - 0.0232 \\
&* A\,G^{2} + 0.0017A\,G\,T + 0.0043A\,T^{2} + 0.0070G^{3} - 0.0201G^{2}T - 0.0037G\,T^{2} + 0.0001T^{3} \\
&+ 0.0002\dot{\gamma}^{2}A^{2} + 0.0002\dot{\gamma}^{2}A\,G + 0.0004\dot{\gamma}^{2}A\,T + 0.0002\dot{\gamma}^{2}G^{2} + 0.0005\dot{\gamma}^{2}G\,T + 0.0002\dot{\gamma}^{2}T^{2} \\
&- 0.0007\dot{\gamma}A^{3} - 0.0012\dot{\gamma}A^{2}G - 0.0022\dot{\gamma}A^{2}T - 0.0005\dot{\gamma}AG^{2} - 0.0058\dot{\gamma}AGT - 0.0016\dot{\gamma}A\,T^{2} - 0.0008\dot{\gamma}G^{3} \\
&- 0.0032\dot{\gamma}G^{2}T \\
&\quad -0.0019\dot{\gamma}GT^{2} - 0.0006\dot{\gamma}T^{3} + 0.0075A^{4} - 0.0204A^{3}G - 0.0702A^{3}T \\
&\qquad - 0.0087A^{2}G^{2} + 0.1972A^{2}G\,T + 0.0270A^{2}T^{2} + 0.0094A\,G^{3} - 0.1859A\,G^{2}T \\
&\qquad + 0.0026A\,G\,T^{2} + 0.0049A\,T^{3} - 0.0065G^{4} + 0.0527G^{3}T \\
&\qquad - 0.0279G^{2}T^{2} + -0.0041G\,T^{3} + 0.0001T^{4} \\
&\quad -0.0019\dot{\gamma}GT^{2} - 0.0006\dot{\gamma}T^{3} + 0.0075A^{4} - 0.0204A^{3}G - 0.0702A^{3}T \\
&\qquad - 0.0087A^{2}G^{2} + 0.1972A^{2}G\,T + 0.0270A^{2}T^{2} + 0.0094A\,G^{3} - 0.1859A\,G^{2}T \\
&\qquad + 0.0026A\,G\,T^{2} + 0.0049A\,T^{3} - 0.0065G^{4} + 0.0527G^{3}T \\
&\qquad - 0.0279G^{2}T^{2} + -0.0041G\,T^{3} + 0.0001T^{4} \\
&\quad -0.0019\dot{\gamma}GT^{2} - 0.0006\dot{\gamma}T^{3} + 0.0075A^{4} - 0.0204A^{3}G - 0.0702A^{3}T \\
&\qquad - 0.0087A^{2}G^{2} + 0.1972A^{2}G\,T + 0.0270A^{2}T^{2} + 0.0094A\,G^{3} - 0.1859A\,G^{2}T \\
&\qquad + 0.0026A\,G\,T^{2} + 0.0049A\,T^{3} - 0.0065G^{4} + 0.0527G^{3}T \\
&\qquad - 0.0279G^{2}T^{2} + -0.0041G\,T^{3} + 0.0001T^{4}
\end{aligned}
\tag{1}
$$

Table S1: Coefficients of all variables for 4th order polynomial predictive model

	coef	std err	t	P>|t|	[0.025	0.975]
const	0.0005	2.95e-05	17.003	0.000	0.000	0.001
x1	-0.0001	2.63e-05	-4.687	0.000	-0.000	-7.12e-05
x2	0.0025	0.000	8.112	0.000	0.002	0.003
x3	0.0003	0.000	1.055	0.294	-0.000	0.001
x4	0.0012	8.15e-05	14.714	0.000	0.001	0.001
x5	4.399e-05	7.32e-06	6.007	0.000	2.95e-05	5.85e-05
x6	-0.0003	6.42e-05	-5.086	0.000	-0.000	-0.000
x7	-0.0003	6.83e-05	-4.554	0.000	-0.000	-0.000
x8	-0.0003	9.12e-05	-3.831	0.000	-0.001	-0.000
x9	0.0079	0.001	5.706	0.000	0.005	0.011
x10	0.0045	0.000	13.919	0.000	0.004	0.005
x11	0.0077	0.001	7.374	0.000	0.006	0.010
x12	-0.0023	0.001	-1.674	0.097	-0.005	0.000
x13	0.0004	0.001	0.387	0.700	-0.002	0.002
x14	0.0015	0.000	14.399	0.000	0.001	0.002
x15	-7.581e-06	9.47e-07	-8.005	0.000	-9.46e-06	-5.7e-06
x16	0.0001	1.46e-05	7.363	0.000	7.86e-05	0.000

x17	0.0001	1.53e-05	7.042	0.000	7.72e-05	0.000
x18	0.0001	3.59e-05	3.815	0.000	6.58e-05	0.000
x19	-0.0007	0.000	-3.704	0.000	-0.001	-0.000
x20	-0.0008	0.000	-2.788	0.006	-0.001	-0.000
x21	-0.0012	0.000	-3.644	0.000	-0.002	-0.001
x22	-0.0006	0.000	-3.357	0.001	-0.001	-0.000
x23	-0.0012	0.000	-3.414	0.001	-0.002	-0.000
x24	-0.0005	0.000	-3.739	0.000	-0.001	-0.000
x25	-0.0075	0.003	-2.467	0.015	-0.014	-0.001
x26	0.0364	0.007	4.910	0.000	0.022	0.051
x27	0.0340	0.007	5.136	0.000	0.021	0.047
x28	-0.0176	0.007	-2.380	0.019	-0.032	-0.003
x29	0.0170	0.002	10.536	0.000	0.014	0.020
x30	0.0102	0.001	7.292	0.000	0.007	0.013
x31	0.0134	0.003	4.422	0.000	0.007	0.019
x32	-0.0143	0.007	-2.160	0.033	-0.027	-0.001
x33	0.0004	0.001	0.313	0.755	-0.002	0.003
x34	0.0017	0.000	14.293	0.000	0.001	0.002
x35	7.389e-07	8.78e-08	8.420	0.000	5.65e-07	9.13e-07
x36	-1.822e-05	2.34e-06	-7.777	0.000	-2.29e-05	-1.36e-05
x37	-1.812e-05	2.32e-06	-7.799	0.000	-2.27e-05	-1.35e-05
x38	-2.431e-05	6.93e-06	-3.507	0.001	-3.8e-05	-1.06e-05
x39	0.0002	2.85e-05	5.896	0.000	0.000	0.000
x40	0.0002	4.51e-05	5.262	0.000	0.000	0.000
x41	0.0005	0.000	3.336	0.001	0.000	0.001
x42	0.0002	3.02e-05	5.471	0.000	0.000	0.000
x43	0.0005	0.000	3.252	0.002	0.000	0.001
x44	0.0002	5.03e-05	3.648	0.000	8.38e-05	0.000
x45	-0.0006	0.000	-2.263	0.026	-0.001	-7.62e-05
x46	-0.0014	0.001	-1.828	0.070	-0.003	0.000
x47	-0.0034	0.001	-2.253	0.026	-0.006	-0.000
x48	-0.0004	0.001	-0.518	0.606	-0.002	0.001
x49	-0.0044	0.003	-1.741	0.084	-0.009	0.001
x50	-0.0016	0.000	-3.536	0.001	-0.002	-0.001
x51	-0.0008	0.000	-2.984	0.004	-0.001	-0.000
x52	-0.0033	0.001	-2.404	0.018	-0.006	-0.001
x53	-0.0016	0.000	-3.329	0.001	-0.003	-0.001
x54	-0.0005	0.000	-3.709	0.000	-0.001	-0.000
x55	0.0112	0.003	4.423	0.000	0.006	0.016

x56	-0.0065	0.006	-1.112	0.269	-0.018	0.005
x57	-0.0650	0.021	-3.038	0.003	-0.107	-0.023
x58	0.0076	0.001	5.408	0.000	0.005	0.010
x59	0.2900	0.065	4.474	0.000	0.162	0.418
x60	0.0471	0.009	5.093	0.000	0.029	0.065
x61	0.0290	0.006	4.982	0.000	0.017	0.041
x62	-0.1774	0.065	-2.747	0.007	-0.305	-0.049
x63	0.0232	0.002	10.295	0.000	0.019	0.028
x64	0.0115	0.002	7.266	0.000	0.008	0.015
x65	-0.0050	0.002	-2.009	0.047	-0.010	-6.81e-05
x66	0.0836	0.021	3.924	0.000	0.041	0.126
x67	-0.0202	0.009	-2.196	0.030	-0.038	-0.002
x68	0.0005	0.002	0.289	0.773	-0.003	0.004
x69	0.0018	0.000	14.248	0.000	0.002	0.002

Table S2: All rules for decision tree-based model.

```
|--- SR <= 5.39
|   |--- SR <= 0.96
|   |   |--- G <= 3.12
|   |   |   |--- SR <= 0.34
|   |   |   |   |--- T <= 0.75
|   |   |   |   |   |--- SR <= 0.15
|   |   |   |   |   |   |--- value: [14.16]
|   |   |   |   |   |--- SR >  0.15
|   |   |   |   |   |   |--- SR <= 0.24
|   |   |   |   |   |   |   |--- value: [13.89]
|   |   |   |   |   |   |--- SR >  0.24
|   |   |   |   |   |   |   |--- value: [13.73]
|   |   |   |   |--- T >  0.75
|   |   |   |   |   |--- SR <= 0.17
|   |   |   |   |   |   |--- A <= 4.50
|   |   |   |   |   |   |   |--- SR <= 0.12
|   |   |   |   |   |   |   |   |--- value: [14.35]
|   |   |   |   |   |   |   |--- SR >  0.12
|   |   |   |   |   |   |   |   |--- value: [14.22]
|   |   |   |   |   |   |--- A >  4.50
|   |   |   |   |   |   |   |--- SR <= 0.12
|   |   |   |   |   |   |   |   |--- value: [14.64]
|   |   |   |   |   |   |   |--- SR >  0.12
|   |   |   |   |   |   |   |   |--- value: [14.49]
|   |   |   |   |   |--- SR >  0.17
|   |   |   |   |   |   |--- G <= 2.50
|   |   |   |   |   |   |   |--- SR <= 0.24
|   |   |   |   |   |   |   |   |--- value: [14.33]
|   |   |   |   |   |   |   |--- SR >  0.24
|   |   |   |   |   |   |   |   |--- value: [14.15]
|   |   |   |   |   |   |--- G >  2.50
|   |   |   |   |   |   |   |--- value: [14.07]
|   |   |   |--- SR >  0.34
|   |   |   |   |--- SR <= 0.68
|   |   |   |   |   |--- G <= 2.12
|   |   |   |   |   |   |--- SR <= 0.48
|   |   |   |   |   |   |   |--- value: [13.95]
|   |   |   |   |   |   |--- SR >  0.48
|   |   |   |   |   |   |   |--- value: [13.74]
|   |   |   |   |   |--- G >  2.12
|   |   |   |   |   |   |--- SR <= 0.48
|   |   |   |   |   |   |   |--- T <= 0.75
|   |   |   |   |   |   |   |   |--- value: [13.57]
|   |   |   |   |   |   |   |--- T >  0.75
|   |   |   |   |   |   |   |   |--- value: [13.72]
|   |   |   |   |   |   |--- SR >  0.48
|   |   |   |   |   |   |   |--- T <= 0.75
|   |   |   |   |   |   |   |   |--- value: [13.39]
|   |   |   |   |   |   |   |--- T >  0.75
|   |   |   |   |   |   |   |   |--- value: [13.52]
|   |   |   |   |--- SR >  0.68
|   |   |   |   |   |--- G <= 2.12
|   |   |   |   |   |   |--- value: [13.53]
|   |   |   |   |   |--- G >  2.12
|   |   |   |   |   |   |   |--- A <= 2.75
|   |   |   |   |   |   |   |   |--- value: [11.98]
|   |   |   |   |   |   |   |--- A >  2.75
|   |   |   |   |   |   |   |   |--- value: [12.18]
|   |   |   |--- T >  0.75
|   |   |   |   |--- SR <= 0.34
|   |   |   |   |   |--- SR <= 0.17
|   |   |   |   |   |   |--- G <= 4.50
|   |   |   |   |   |   |   |--- value: [13.58]
|   |   |   |   |   |   |--- G >  4.50
|   |   |   |   |   |   |   |--- SR <= 0.12
|   |   |   |   |   |   |   |   |--- value: [14.03]
|   |   |   |   |   |   |   |--- SR >  0.12
|   |   |   |   |   |   |   |   |--- value: [13.82]
|   |   |   |   |   |--- SR >  0.17
|   |   |   |   |   |   |--- SR <= 0.24
|   |   |   |   |   |   |   |--- G <= 4.50
|   |   |   |   |   |   |   |   |--- value: [13.41]
|   |   |   |   |   |   |   |--- G >  4.50
|   |   |   |   |   |   |   |   |--- value: [13.59]
|   |   |   |   |   |   |--- SR >  0.24
|   |   |   |   |   |   |   |--- value: [13.35]
|   |   |   |   |--- SR >  0.34
|   |   |   |   |   |--- SR <= 0.48
|   |   |   |   |   |   |--- G <= 4.50
|   |   |   |   |   |   |   |--- value: [13.04]
|   |   |   |   |   |   |--- G >  4.50
|   |   |   |   |   |   |   |--- value: [13.10]
|   |   |   |   |   |--- SR >  0.48
|   |   |   |   |   |   |--- SR <= 0.68
|   |   |   |   |   |   |   |--- value: [12.84]
|   |   |   |   |   |   |--- SR >  0.68
|   |   |   |   |   |   |   |--- A <= 2.50
|   |   |   |   |   |   |   |   |--- value: [12.60]
|   |   |   |   |   |   |   |--- A >  2.50
|   |   |   |   |   |   |   |   |--- value: [12.64]
|   |--- SR >  0.96
|   |   |--- G <= 3.12
|   |   |   |--- SR <= 1.91
|   |   |   |   |--- A <= 5.12
|   |   |   |   |   |--- SR <= 1.35
|   |   |   |   |   |   |--- G <= 2.50
|   |   |   |   |   |   |   |--- value: [13.24]
|   |   |   |   |   |   |--- G >  2.50
|   |   |   |   |   |   |   |--- value: [13.11]
|   |   |   |   |   |--- SR >  1.35
|   |   |   |   |   |   |--- value: [12.98]
|   |   |   |   |--- A >  5.12
|   |   |   |   |   |--- SR <= 1.35
|   |   |   |   |   |   |--- value: [12.98]
|   |   |   |   |   |--- SR >  1.35
|   |   |   |   |   |   |--- value: [12.78]
|   |   |   |--- SR >  1.91
|   |   |   |   |--- SR <= 3.81
|   |   |   |   |   |   |   |   |   |--- value: [12.03]
|   |   |   |   |   |--- SR >  1.91
|   |   |   |   |   |   |--- value: [11.75]
|   |   |   |--- SR >  2.70
|   |   |   |   |--- G <= 5.12
|   |   |   |   |   |--- SR <= 3.81
|   |   |   |   |   |   |--- G <= 4.12
|   |   |   |   |   |   |   |--- A <= 3.62
|   |   |   |   |   |   |   |   |--- value: [11.73]
|   |   |   |   |   |   |   |--- A >  3.62
|   |   |   |   |   |   |   |   |--- value: [11.56]
|   |   |   |   |   |   |--- G >  4.12
|   |   |   |   |   |   |   |--- A <= 2.62
|   |   |   |   |   |   |   |   |--- value: [11.47]
|   |   |   |   |   |   |   |--- A >  2.62
|   |   |   |   |   |   |   |   |--- value: [11.45]
|   |   |   |   |   |--- SR >  3.81
|   |   |   |   |   |   |--- A <= 2.50
|   |   |   |   |   |   |   |--- value: [11.18]
|   |   |   |   |   |   |--- A >  2.50
|   |   |   |   |   |   |   |--- T <= 0.75
|   |   |   |   |   |   |   |   |--- value: [11.37]
|   |   |   |   |   |   |   |--- T >  0.75
|   |   |   |   |   |   |   |   |--- value: [11.49]
|   |   |   |   |--- G >  5.12
|   |   |   |   |   |--- SR <= 3.81
|   |   |   |   |   |   |--- value: [11.14]
|   |   |   |   |   |--- SR >  3.81
|   |   |   |   |   |   |--- value: [10.91]
|--- SR >  5.39
|   |--- SR <= 42.80
|   |   |--- SR <= 15.20
|   |   |   |--- G <= 4.12
|   |   |   |   |--- G <= 3.12
|   |   |   |   |   |--- SR <= 7.61
|   |   |   |   |   |   |--- A <= 4.62
|   |   |   |   |   |   |   |--- value: [11.92]
|   |   |   |   |   |   |--- A >  4.62
|   |   |   |   |   |   |   |--- value: [11.69]
|   |   |   |   |   |--- SR >  7.61
|   |   |   |   |   |   |--- SR <= 10.76
|   |   |   |   |   |   |   |--- A <= 5.12
|   |   |   |   |   |   |   |   |--- A <= 4.50
|   |   |   |   |   |   |   |   |   |--- value: [11.67]
|   |   |   |   |   |   |   |   |--- A >  4.50
|   |   |   |   |   |   |   |   |   |--- value: [11.64]
|   |   |   |   |   |   |   |--- A >  5.12
|   |   |   |   |   |   |   |   |--- value: [11.41]
|   |   |   |   |   |   |--- SR >  10.76
|   |   |   |   |   |   |   |--- G <= 2.50
|   |   |   |   |   |   |   |   |--- value: [11.34]
|   |   |   |   |   |   |   |--- G >  2.50
|   |   |   |   |   |   |   |   |--- value: [11.42]
|   |   |   |   |--- G >  3.12
|   |   |   |   |   |   |--- SR <= 21.45
|   |   |   |   |   |   |   |--- G <= 4.12
|   |   |   |   |   |   |   |   |--- A <= 3.62
|   |   |   |   |   |   |   |   |   |--- value: [10.50]
|   |   |   |   |   |   |   |   |--- A >  3.62
|   |   |   |   |   |   |   |   |   |--- value: [10.52]
|   |   |   |   |   |   |   |--- G >  4.12
|   |   |   |   |   |   |   |   |--- value: [10.38]
|   |   |   |   |   |   |--- SR >  21.45
|   |   |   |   |   |   |   |--- A <= 3.62
|   |   |   |   |   |   |   |   |--- value: [10.25
|   |   |   |   |   |   |   |--- A >  3.62
|   |   |   |   |   |   |   |   |--- value: [10.29]
|   |   |   |   |   |--- SR >  30.30
|   |   |   |   |   |   |--- G <= 4.12
|   |   |   |   |   |   |   |--- A <= 3.62
|   |   |   |   |   |   |   |   |--- value: [9.98]
|   |   |   |   |   |   |   |--- A >  3.62
|   |   |   |   |   |   |   |   |--- value: [10.05]
|   |   |   |   |   |   |--- G >  4.12
|   |   |   |   |   |   |   |--- value: [9.89]
|   |   |   |--- G >  4.62
|   |   |   |   |--- SR <= 21.45
|   |   |   |   |   |--- G <= 5.12
|   |   |   |   |   |   |--- value: [10.03]
|   |   |   |   |   |--- G >  5.12
|   |   |   |   |   |   |--- value: [9.93]
|   |   |   |   |--- SR >  21.45
|   |   |   |   |   |--- SR <= 30.30
|   |   |   |   |   |   |--- A <= 2.12
|   |   |   |   |   |   |   |--- value: [9.75]
|   |   |   |   |   |   |--- A >  2.12
|   |   |   |   |   |   |   |--- value: [9.66]
|   |   |   |   |   |--- SR >  30.30
|   |   |   |   |   |   |--- value: [9.45]
|   |--- SR >  42.80
|   |   |--- SR <= 85.40
|   |   |   |--- G <= 4.62
|   |   |   |   |--- SR <= 60.45
|   |   |   |   |   |--- G <= 3.12
|   |   |   |   |   |   |--- G <= 2.62
|   |   |   |   |   |   |   |--- value: [9.87]
|   |   |   |   |   |   |--- G >  2.62
|   |   |   |   |   |   |   |--- value: [10.03]
|   |   |   |   |   |--- G >  3.12
|   |   |   |   |   |   |--- A <= 3.75
|   |   |   |   |   |   |   |--- value: [9.63]
|   |   |   |   |   |   |--- A >  3.75|   |   |   |   |   |   |   |--- value: [9.76]
|   |   |   |   |--- SR >  60.45
|   |   |   |   |   |--- G <= 3.12
|   |   |   |   |   |   |--- T <= 0.75
|   |   |   |   |   |   |   |--- value: [9.51]
|   |   |   |   |   |   |--- T >  0.75
|   |   |   |   |   |   |   |--- value: [9.62]
```

```
| | | | | | |--- T <= 0.75
| | | | | | | |--- value: [13.19]
| | | | | | |--- T > 0.75
| | | | | | | |--- value: [13.31]
| | |--- G > 3.12
| | | |--- T <= 0.75
| | | | |--- SR <= 0.34
| | | | | |--- SR <= 0.17
| | | | | | |--- SR <= 0.12
| | | | | | | |--- G <= 4.75
| | | | | | | | |--- value: [12.95]
| | | | | | | |--- G > 4.75
| | | | | | | | |--- value: [12.98]
| | | | | | |--- SR > 0.12
| | | | | | | |--- A <= 3.75
| | | | | | | | |--- value: [12.86]
| | | | | | | |--- A > 3.75
| | | | | | | | |--- value: [12.86]
| | | | | |--- SR > 0.17
| | | | | | |--- SR <= 0.24
| | | | | | | |--- G <= 4.75
| | | | | | | | |--- G <= 3.75
| | | | | | | | | |--- value: [12.77]
| | | | | | | | |--- G > 3.75
| | | | | | | | | |--- value: [12.74]
| | | | | | | |--- G > 4.75
| | | | | | | | |--- value: [12.68]
| | | | | | |--- SR > 0.24
| | | | | | | |--- A <= 2.75
| | | | | | | | |--- value: [12.52]
| | | | | | | |--- A > 2.75
| | | | | | | | |--- value: [12.62]
| | | | |--- SR > 0.34
| | | | | |--- SR <= 0.68
| | | | | | |--- A <= 2.75
| | | | | | | |--- SR <= 0.48
| | | | | | | | |--- value: [12.35]
| | | | | | | |--- SR > 0.48
| | | | | | | | |--- value: [12.17]
| | | | | | |--- A > 2.75
| | | | | | | |--- SR <= 0.48
| | | | | | | | |--- value: [12.48]
| | | | | | | |--- SR > 0.48
| | | | | | | | |--- A <= 3.75
| | | | | | | | | |--- value: [12.34]
| | | | | | | | |--- A > 3.75
| | | | | | | | | |--- value: [12.41]
| | | | | |--- SR > 0.68
```

```
| | | | | |--- SR <= 2.70
| | | | | | |--- value: [12.51]
| | | | | |--- SR > 2.70
| | | | | | |--- A <= 5.12
| | | | | | | |--- value: [12.43]
| | | | | | |--- A > 5.12
| | | | | | | |--- value: [12.24]
| | | | |--- SR > 3.81
| | | | | |--- T <= 0.75
| | | | | | |--- value: [11.95]
| | | | | |--- T > 0.75
| | | | | | |--- A <= 4.50
| | | | | | | |--- value: [12.17]
| | | | | | |--- A > 4.50
| | | | | | | |--- value: [12.17]
| | |--- G > 3.12
| | | |--- SR <= 2.70
| | | | |--- T <= 0.75
| | | | | |--- A <= 3.75
| | | | | | |--- SR <= 1.35
| | | | | | | |--- value: [11.79]
| | | | | | |--- SR > 1.35
| | | | | | | |--- G <= 4.75
| | | | | | | | |--- SR <= 1.91
| | | | | | | | | |--- value: [11.84]
| | | | | | | | |--- SR > 1.91
| | | | | | | | | |--- value: [11.64]
| | | | | | | |--- G > 4.75
| | | | | | | | |--- value: [11.58]
| | | | | |--- A > 3.75
| | | | | | |--- SR <= 1.35
| | | | | | | |--- value: [12.09]
| | | | | | |--- SR > 1.35
| | | | | | | |--- SR <= 1.91
| | | | | | | | |--- value: [11.92]
| | | | | | | |--- SR > 1.91
| | | | | | | | |--- value: [11.76]
| | | | |--- T > 0.75
| | | | | |--- SR <= 1.91
| | | | | | |--- SR <= 1.35
| | | | | | | |--- A <= 2.50
| | | | | | | | |--- value: [12.30]
| | | | | | | |--- A > 2.50
| | | | | | | | |--- value: [12.43]
| | | | | | |--- SR > 1.35
| | | | | | | |--- G <= 4.50
| | | | | | | | |--- value: [12.19]
| | | | | | | |--- G > 4.50
```

```
| | | | | |--- SR <= 7.61
| | | | | | |--- A <= 3.62
| | | | | | | |--- value: [11.25]
| | | | | | |--- A > 3.62
| | | | | | | |--- value: [11.17]
| | | | | |--- SR > 7.61
| | | | | | |--- value: [10.96]
| | | |--- G > 4.12
| | | | |--- A <= 2.75
| | | | | |--- SR <= 7.61
| | | | | | |--- value: [10.68]
| | | | | |--- SR > 7.61| | | | | |
|--- A <= 2.12
| | | | | | | |--- value: [10.31]
| | | | | | |--- A > 2.12
| | | | | | | |--- value: [10.43]
| | | | |--- A > 2.75
| | | | | |--- SR <= 10.76
| | | | | | |--- SR <= 7.61
| | | | | | | |--- value: [11.05]
| | | | | | |--- SR > 7.61
| | | | | | | |--- value: [10.84]
| | | | | |--- SR > 10.76
| | | | | | |--- value: [10.61]
| | |--- SR > 15.20
| | | |--- G <= 3.12
| | | | |--- SR <= 30.30| | | | | |---
SR <= 21.45
| | | | | | |--- T <= 0.75
| | | | | | | |--- value: [10.87]
| | | | | | |--- T > 0.75
| | | | | | | |--- value: [11.16]
| | | | | |--- SR > 21.45
| | | | | | |--- G <= 2.62
| | | | | | | |--- value: [10.59]
| | | | | | |--- G > 2.62
| | | | | | | |--- value: [10.87]
| | | | |--- SR > 30.30
| | | | | |--- G <= 2.62
| | | | | | |--- G <= 2.12
| | | | | | | |--- value: [10.36]
| | | | | | |--- G > 2.12
| | | | | | | |--- value: [10.26]
| | | | | |--- G > 2.62
| | | | | | |--- value: [10.54]
| | | |--- G > 3.12
| | | | |--- G <= 4.62
| | | | | |--- SR <= 30.30
```

```
| | | | | |--- G > 3.12
| | | | | | |--- G <= 4.12
| | | | | | | |--- A <= 3.62
| | | | | | | | |--- value: [9.44]
| | | | | | | |--- A > 3.62
| | | | | | | | |--- value: [9.39]
| | | | | | |--- G > 4.12
| | | | | | | |--- value: [9.31]
| | | |--- G > 4.62
| | | | |--- SR <= 60.45
| | | | | |--- G <= 5.12
| | | | | | |--- value: [9.14]
| | | | | |--- G > 5.12
| | | | | | |--- value: [9.11]
| | | | |--- SR > 60.45
| | | | | |--- value: [8.83]
| | |--- SR > 85.40
| | | |--- A <= 2.62
| | | | |--- T <= 0.75
| | | | | |--- value: [8.51]
| | | | |--- T > 0.75
| | | | | |--- value: [8.55]
| | | |--- A > 2.62
| | | | |--- T <= 0.75
| | | | | |--- A <= 4.75
| | | | | | |--- G <= 3.75
| | | | | | | |--- value: [8.90]
| | | | | | |--- G > 3.75
| | | | | | | |--- value: [8.95]
| | | | | |--- A > 4.75
| | | | | | |--- value: [9.06]
| | | | |--- T > 0.75
| | | | | |--- A <= 3.50
| | | | | | |--- value: [9.08]
| | | | | |--- A > 3.50
| | | | | | |--- A <= 4.50
| | | | | | | |--- value: [9.19]
| | | | | | |--- A > 4.50
| | | | | | | |--- value: [9.08
```
